# Integrating clinical decision support systems for pharmacogenomic testing into clinical routine - a scoping review of designs of user-system interactions in recent system development

**DOI:** 10.1186/s12911-017-0480-y

**Published:** 2017-06-06

**Authors:** Marc Hinderer, Martin Boeker, Sebastian A. Wagner, Martin Lablans, Stephanie Newe, Jan L. Hülsemann, Michael Neumaier, Harald Binder, Harald Renz, Till Acker, Hans-Ulrich Prokosch, Martin Sedlmayr

**Affiliations:** 1 Medical Informatics, Friedrich-Alexander-Universität Erlangen-Nürnberg, Wetterkreuz 13, 91058 Erlangen, Germany; 2 Medical Informatics, Institute of Medical Biometry and Statistics, Faculty of Medicine and Medical Center - University of Freiburg, Freiburg, Germany; 3 Department of Medicine, Hematology/Oncology, Goethe University, Frankfurt, Germany; 4 Medical Informatics in Translational Oncology, German Cancer Research Center, Heidelberg, Germany; 5 University hospital Magdeburg, Magdeburg, Germany; 6 Institute for Clinical Chemistry, Medical Faculty Mannheim, Ruprecht-Karls-University Heidelberg, Mannheim, Germany; 7 Institute of Medical Biostatistics, Epidemiology and Informatics (IMBEI), University Medical Center of the Johannes Gutenberg University Mainz, Mainz, Germany; 8 University of Marburg, Institute of Laboratory Medicine, Marburg, Germany; 9 Institute of Neuropathology, University of Giessen, Giessen, Germany

**Keywords:** Pharmacogenomic, Clinical decision support, User-system interaction, Developments, Precision medicine

## Abstract

**Background:**

Pharmacogenomic clinical decision support systems (CDSS) have the potential to help overcome some of the barriers for translating pharmacogenomic knowledge into clinical routine. Before developing a prototype it is crucial for developers to know which pharmacogenomic CDSS features and user-system interactions have yet been developed, implemented and tested in previous pharmacogenomic CDSS efforts and if they have been successfully applied. We address this issue by providing an overview of the designs of user-system interactions of recently developed pharmacogenomic CDSS.

**Methods:**

We searched PubMed for pharmacogenomic CDSS published between January 1, 2012 and November 15, 2016. Thirty-two out of 118 identified articles were summarized and included in the final analysis. We then compared the designs of user-system interactions of the 20 pharmacogenomic CDSS we had identified.

**Results:**

Alerts are the most widespread tools for physician-system interactions, but need to be implemented carefully to prevent alert fatigue and avoid liabilities. Pharmacogenomic test results and override reasons stored in the local EHR might help communicate pharmacogenomic information to other internal care providers. Integrating patients into user-system interactions through patient letters and online portals might be crucial for transferring pharmacogenomic data to external health care providers. Inbox messages inform physicians about new pharmacogenomic test results and enable them to request pharmacogenomic consultations. Search engines enable physicians to compare medical treatment options based on a patient’s genotype.

**Conclusions:**

Within the last 5 years, several pharmacogenomic CDSS have been developed. However, most of the included articles are solely describing prototypes of pharmacogenomic CDSS rather than evaluating them. To support the development of prototypes further evaluation efforts will be necessary. In the future, pharmacogenomic CDSS will likely include prediction models to identify patients who are suitable for preemptive genotyping.

**Electronic supplementary material:**

The online version of this article (doi:10.1186/s12911-017-0480-y) contains supplementary material, which is available to authorized users.

## Background

Genetic variants can influence drug metabolism, transport and receptor response and thereby lead to reduced drug activity or increased toxicity [1–3]. Prominent examples are the anticoagulants clopidogrel and warfarin that are metabolized by CYP2C19 and CYP2C9, respectively. Variants in these enzymes can alter the plasma levels of the anticoagulants and thereby lead to insufficient anticoagulation or increased risk of bleeding. The influence of genetic variants on drug activity led to the development of pharmacogenomic tests and drug dosing guidelines which incorporate pharmacogenomic data into the drug prescription process [4, 5]. An example for the development of pharmacogenomic recommendations and best practices guidelines is the publicly available web-based knowledge base PharmGKB (https://www.pharmgkb.org/). It includes dosing guidelines by the Clinical Pharmacogenetics Implementation Consortium (CPIC), the Royal Dutch Association for the Advancement of Pharmacy - Pharmacogenetics Working Group (DPWG), the Canadian Pharmacogenomics Network for Drug Safety (CPNDS) and other professional society. Other examples of pharmacogenomic knowledge bases are the OncoKB (oncokb.org/#/) by the Memorial Sloan Kettering Cancer Center and the PMKB (https://pmkb.weill.cornell.edu/) by the Weil Cornell Medical College.

In prospect of whole genome sequencing, the discovery of new gene-drug interaction pairs is very likely and will further increase the pharmacogenomic knowledge base. However, translating this pharmacogenomic knowledge into clinical routine has been slow and is hindered by the lack of the physicians’ knowledge and experience in pharmacogenomic testing [1, 6–8].

In recent years, informatics has gained crucial relevance for improving patient care. This includes a considerable amount of published literature which describes the current efforts on developing and implementing pharmacogenomic clinical decision support systems (CDSS) [9–11]. Pharmacogenomic CDSS might help overcome some of the barriers of implementing pharmacogenomic knowledge into clinical routine [7, 10].

Pharmacogenomic CDSS are computer-based systems which support health care providers in prescribing drugs at the point of care. These systems provide physicians and other health care providers with reasonably filtered pharmacogenomic information such as gene-drug interaction alerts or patient-specific treatment recommendations. A pharmacogenomic CDSS can either be integrated into the local hospital information system (HIS) or used as a separate program such as a web service or mobile application [10]. Furthermore, pharmacogenomic CDSS can provide passive or active clinical decision support (CDS). Active CDS include rules and alerts. An alert, for example, might be triggered because a high-risk drug is prescribed and pharmacogenomic testing prior to the drug application would be indicated. Passive CDS require the user to actively search for the information, e.g. clicking on a button or opening a case report [10, 12].

To develop a prototype it is crucial for developers to know which pharmacogenomic CDSS features and user-system interactions have been developed, implemented and tested in previous pharmacogenomic CDSS efforts and if they were successfully applied. Welch and Kawamoto systematically reviewed the literature on pharmacogenomic CDSS including manuscripts from 1990 to 2011 [13]. Given the recent rise of omics technologies, the findings of their systematic review cannot include the most recent developments of pharmacogenomic CDSS. In addition to that, Welch and Kawamoto did not compare the designs of user-system interactions (e.g. passive vs. active CDS, displaying pre-testing vs. post-testing alerts, or the contents of such alerts presented to the user).

Dunnenberger et al. and Hicks et al. previously reported on recent developments of pharmacogenomic CDSS since 2012 [14, 15]. They also mentioned some potential concepts for implementing pharmacogenomic CDSS into clinical routine. However, they did neither analyze designs of user-system interactions nor did they describe whether or not such designs have been evaluated. Furthermore, they limited their scope to concepts involving an EHR. For developers, it is crucial to also know which potential designs of user-system interactions exist without involving an EHR.

To our knowledge, there is no systematic or scoping review to date which provides an overview of the recent developments of pharmacogenomic CDSS and their designs of user-system interactions. Given the clinical importance of pharmacogenomic CDSS, we address this topic by comparing the functionalities and the designs of user-system interactions of published pharmacogenomic CDSS since 2012. The objective of this paper is to provide an overview of the recent developments of pharmacogenomic CDSS and their designs of user-system interactions.

## Methods

To minimize bias in the selection of included studies, we followed the Preferred Reporting Items for Systematic Reviews and Meta-Analysis (PRISMA) guidelines as far as appropriate for this scoping review [16, 17]. We achieved a high degree of completeness by providing information on 22 out of the 27 points recommended (see Additional file 1). A study protocol was written prior to the investigation, but has not been registered.

### Information sources and inclusion criteria

We searched PubMed for pharmacogenomic CDSS published between January 1, 2012 and November 30, 2016. We used the keywords “pharmacogenetic*”, “pharmacogenomic*”, “decision support” and “medical decision making” for the search query as shown in Table 1. The final search was conducted on December 01, 2016. The inclusion criteria for the scoping review were as follows: English article; manuscript in peer-reviewed journal; research article; describing a clinical prototype or a fully developed pharmacogenomic CDSS in clinical routine; describing the functionalities and the design of user-system interactions of a pharmacogenomic CDSS.

**Table 1:** PubMed search query

PubMed search query	
1. Pharmacogenetics[MeSH Terms] 2. pharmacogenetic*[tw] 3. pharmacogenomic*[tw] 4. “decision support systems, clinical”[MeSH Terms] 5. “decision support”[tw] 6. “decision making”[tw] 7. (“2012/01/01”[PDAT] : “2016/11/30”[PDAT]) 8. #1 OR #2 OR #3 9. #4 OR #5 OR #6 10. #7 AND #8 AND #9	

Two authors (MH and MS) screened the titles, index terms, and abstracts for all identified publications to determine, if all inclusion requirements were met. This was done independently by both authors (MH and MS) and for all identified articles. In this way, potential differences in the judgment of including or excluding certain articles could be spotted. If no clear decision could be made on the basis of this information, the article was obtained in full-text and a decision on the inclusion was based on information from the full-text. The full-texts of all included articles were obtained via institutional library access or the authors’ user profile on the ResearchGate platform.

### Data abstraction

For all publications meeting the inclusion criteria listed above, the following data items were extracted by both authors (MH and MS) independently and for all included articles: system or project name; users and study location; CDSS development status; EHR integration; web-based access; active or passive CDS and the CDS features for user-system interactions (categorized as “alerts”, “reports”, “EHR data”, “inbox messages”, “search engines” and “others”). Differences were discussed among co-authors (MH, MB, MS) in order to resolve disagreements and to achieve a consensus. Following this, all abstracted data were reviewed and revised by all co-authors, which led to the final version of data abstraction of all articles.

The system or project name was either the specific pharmacogenomic CDSS name (if applicable) or (if no system name existed) the related project name. If neither a system nor a project name was available, we created a surrogate pharmacogenomic CDSS name (marked with s/n for “surrogate name”). Users were defined as physicians, pharmacists, other health care providers or patients. The study location was the main institution, where research was conducted. The CDSS development status was either prototype or fully developed. The EHR integration category distinguished between pharmacogenomic CDSS which were integrated into an electronic health record (EHR) or a computerized physician order entry system (CPOE) (“yes”) and those designed as stand-alone systems (“no”). A stand-alone system exists in parallel to the EHR or the CPOE. If the pharmacogenomic CDSS was accessible through the internet (e.g. online portals of laboratories), it was marked as “web-based access”. The alert category defined whether the pharmacogenomic CDSS provided pre-testing or post-testing alerts and which information these alerts contained. A report was defined as a summary report or patient letters including the patients’ pharmacogenomic information. Within the EHR data category every kind of pharmacogenomic information stored in the EHR for clinical decision support was included. Furthermore, inbox message designs and search engine designs used for pharmacogenomic CDSS were documented if applicable.

The risk of bias for individual studies was not systematically assessed.

### Data analysis and presentation

Extracted and categorized data were used and the data items were grouped into two logical domains. The first domain included the categories “system or project name”, “users and study location” and “CDSS development status“. Whereas the second domain comprised the categories specifying the user-system interaction: “EHR integration”, “web-based access”, “active or passive CDS”, “alerts”, “reports”, “EHR data”, “inbox messages”, “search engines” and “others“. We summarized the articles in the form of tables and narrative discussion. Differences were discussed among co-authors (MH, MS) in order to resolve disagreements and to achieve a consensus. Following this, the results by MH and MS were reviewed and consented by all co-authors. This led to the final interpretation and presentation of the abstracted data of all articles.

To provide an unbiased overview of all pharmacogenomic CDSS including those mentioned in this scoping review, we grouped all articles which used a common pharmacogenomic CDSS. The articles were grouped by the abstracted system or project names and by the attributes of the first domain.

Furthermore, we identified and analyzed the designs of user-system interactions of recently published pharmacogenomic CDSS. Therefore, we used the abstracted system or project names and the results of the tabulated attributes of the second domain.

## Results

**Fig. 1:**
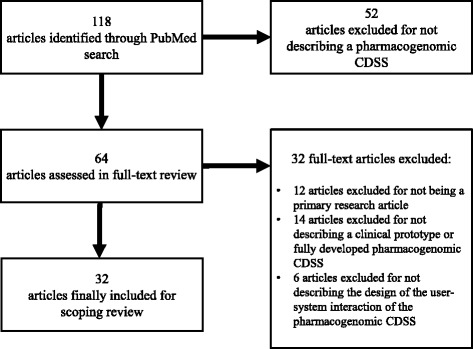
PubMed search and process of article selection. CDSS: clinical decision support system

The PubMed search identified 118 potentially relevant articles. During the title and abstract review, 54 articles were excluded for not describing a pharmacogenomic CDSS. The remaining 64 articles underwent full-text review, after which 12 articles were excluded for not being primary research articles. Furthermore, 14 articles were excluded for not describing a clinical prototype or a fully developed pharmacogenomic CDSS and 12 articles for not being a primary research article. Six out of the remaining 38 publications were excluded for not describing the design of the user-system interaction of their pharmacogenomic CDSS (Fig. 1).

The 32 publication which had been included were grouped by the fact of whether or not they used a common pharmacogenomic CDSS (first domain). Regarding the articles which reported on the RIGHT project and the Personalized Medication Program (PMP) we could not find any information on whether or not they used a common pharmacogenomic CDSS. We categorized the pharmacogenomic CDSS by the “Right Drug, Right Dose, Right Time-Using Genomic Data to Individualize Treatment (RIGHT)” project (three) and the Personalized Medication Program (PMP) (two) into five separate and independent systems (Table 2).

**Table 2:** Selection of 20 pharmacogenomic CDSS

System or project name	Users and study location	Development status	Citation
Clinical Pharmacogenomic Service (CPS)	Boston Children’s Hospital (BCH); Physicians Pharmacists	prototype	Fusaro et al. 2013 [34]; Manzi et al. 2016 [28]
Medicine Safety Code (MSC)	University of Vienna; Physicians and Patients	prototype	Minarro-Gimenez et al. 2014 [18]; Blagec et al. 2016 [10]
UW-PowerChart® prototype (s/n)	University of Washington; Physicians	prototype	Overby et al. 2012 [37]; Devine et al. 2014 [38]; Overby et al. 2015 [11]; Nishimura et al. 2015 [32]; Nishimura et al. 2016 [33]
CU-case series study (s/n)	Columbia University; Physicians	fully developed (CLIA-certified laboratory)	Cabrera und Finkelstein 2012 [26]; Finkelstein et al. 2016 [27]
RIGHT (No.1)	Mayo Clinic; Physicians	prototype	Caraballo et al. 2016 [29]
RIGHT (No. 2)	Mayo Clinic; Physicians	prototype	Vitek et al. 2015 [35]
RIGHT (No. 3)	Mayo Clinic; Physicians	prototype	Ji et al. 2016 [25]
PREDICT	Vanderbilt University; Physicians and Pharmacists	prototype	Pulley et al. 2012 [19]; Peterson et al. 2013 [20]
Personalized Medication Program (PMP) (No. 1)	Cleveland Clinc (Ohio); physicians	prototype	Hicks et al. 2016 [30]; Teng et al. 2014 [52]
Personalized Medication Program (PMP) (No. 2)	Cleveland Clinc (University of Florida) physicians	prototype	Owusu-Obeng et al. 2014 [36]
PG4KDS	St. Jude Children Research Hospital; Physician; Nurse practitioner; Pharmacists; Patients	prototype	Bell et al. 2014 [12]; Hoffman et al. 2014 [39]; Gammal et al. 2016 [31]
GeneSight	No study location; Physicians	fully developed (CLIA-certified laboratory)	Altar et al. 2015 [21]
Genomic prescribing system (GPS) portal	University of Chicago; Physicians; Patients	prototype	O’Donnell et al. 2014 [22]; Hussain et al. 2016 [23]
CLIPMERGE PGx	Mount Sinai Medical Center; Physcians	prototype	Gottesman et al. 2013 [8]
Pharmacogenetics Testing Implementation Committee (PGTIC) (s/n)	National Institutes of Health Clinical Center, Bethesda, Maryland	prototype	Goldspiel et al. 2014 [9]
genAP	Oslo University; Physicians	prototype	Laerum et al. 2013 [40]
YouScript	University of Utah; Physicians; Pharmacists	fully developed	Brixner et al. 2015 [41]
TreatGx	University of British Columbia; primary care physicians; pharmacist	fully developed	Dawes et al. 2016 [43]
Coriell Personalized Medicine Collaborative (CPMC) (s/n)	Ohio State University Wexner Medical Center; patients	prototype	Sweet et al. 2014 [42]
Warfarin Dosing prototype (s/n)	Wishard Hospital and Veterans Affairs Medical Center Indianapolis; Physicians; Pharmacists	prototype	Melton et al. 2016 [24]

Notes: “s/n” = surrogate name

As a result we included 31 research articles which have been published since 2012 and which describe the design of the user-system interactions of 20 different pharmacogenomic CDSS (Table 3). An additional spreadsheet file shows the features of the 20 pharmacogenomic CDSS in more detail (see Additional file 2). A further comparison can be found below (second domain).

**Table 3:** Designs of user-system interactions of the 20 selected pharmacogenomic CDSS

System or project name	CDSS integration	Web-based access	Active or passive CDS	Included features
Clinical Pharmacogenomic Service (CPS)	yes	yes	active; passive	•pre-testing and post-testing alerts •reports •EHR data
Medicine Safety Code (MSC)	no	yes	passive	•reports •search engine •others (interface to upload genetic files; printed QR codes)
UW-PowerChart® prototype (s/n)	yes	no	active	•post-testing alerts •reports •EHR data
CU-case series study (s/n)	no	yes	passive	•reports
RIGHT (No.1)	yes	no	active	•pre-testing and post-testing alerts •EHR data •inbox messages
RIGHT (No. 2)	yes	no	active	•post-testing alerts •EHR data •inbox messages
RIGHT (No. 3)	yes	yes	active	•post-testing alerts •others (online portal for patients)
PREDICT	yes	yes	active; passive	•pre-testing and post-testing alerts •reports •EHR data •others (online portal for patients)
Personalized Medication Program (PMP) (No. 1)	yes	no	active; passive	•pre-testing and post-testing alerts •reports •EHR data •others (virtual PGx consult)
Personalized Medication Program (PMP) (No. 2)	yes	no	active	•post-testing alerts •inbox messages
PG4KDS	yes	no	active	•pre-testing and post-testing alerts •reports •EHR data •inbox messages
GeneSight	no	yes	passive	•reports
Genomic prescribing system (GPS) portal	no	yes	active; passive	•post-testing alerts •reports •search engine •others (virtual PGx consult)
CLIPMERGE PGx	yes	no	active	•post-testing alerts
Pharmacogenetics Testing Implementation Committee (PGTIC) (s/n)	yes	no	active	•pre-testing and post-testing alerts •EHR data •inbox messages
genAP	yes	no	passive	•reports
YouScript	no	no	passive	•reports
TreatGx	yes	no	passive	•EHR data •inbox messages
Coriell Personalized Medicine Collaborative (CPMC) (s/n)	no	yes	passive	•reports •inbox messages
Warfarin Dosing prototype (s/n)	yes	no	active	•post-test alerts

Notes: “s/n” = surrogate name

### EHR integration, web-based access and active or passive CDS

We identified 14 pharmacogenomic CDSS, which were integrated into the local EHR and six systems which were designed as stand-alone systems. Seven pharmacogenomic CDSS provided a web-based access to the pharmacogenomic test results in a password protected online portal for physicians [10, 18–24] or patients [25–27]. 13 pharmacogenomic CDSS provided an active CDSS and 11 CDSS provided a passive CDSS.

### Alerts

#### Designs

In 13 pharmacogenomic CDSS physicians were provided with actionable real-time alerts for gene-based drug prescription at the point of care. This included pre-test alerts in six pharmacogenomic CDSS and post-test alerts in all 13 CDSS. Pre-test alerts were presented when a physician ordered a drug for a patient for whom there was no pharmacogenomic test result in the EHR or who was flagged by a prognostic model for pre-emptive genotyping. For instance, in the PREDICT project at the Vanderbilt University, the prognostic model identified patients with high risk of initiating simvastatin, warfarin or clopidogrel therapy within 3 years [20]. When a high-risk medication is ordered for a patient with a relevant pharmacogenomic test result in the EHR, a post-test alert is presented to the physician to guide prescription.

The pre-test alerts of three pharmacogenomic CDSS presented the clinical impact of a potential drug-gene interaction to the physician [9, 12, 20, 28–31]. Furthermore, all six pharmacogenomic CDSS with pre-test alerts included the recommendation that a specific genotype should be obtained before prescribing the intended drug if potential drug-gene interactions were known. One of them [31] recommended consulting a clinical pharmacist for further treatment evaluation. Five out of the six pharmacogenomic CDSS offered an option to order an appropriate pharmacogenomic test [9, 12, 20, 29–31].

The prescriber could override the pre-test alert in four pharmacogenomic CDSS [9, 12, 29–31] and continue with the order or cancellation of the prescription [9, 12, 31]. If the physician continued with the order, a second screen prompted the physician in one pharmacogenomic CDSS [9] to enter a pre-defined reason for overriding the alert.

Links to further information were provided by four pharmacogenomic CDSS with pre-test alerts [9, 12, 29–31]. Each pre-test alert of these four pharmacogenomic CDSS also referred physicians to further resources, so that physicians could learn more about the relevance of pharmacogenomic results for drugs with potential drug-gene interactions.

In addition, the test status was provided in the order set form in one pharmacogenomic CDSS [9] if the pharmacogenomic result was present, absent, pending, or if the test was not ordered and the date of the last related test was ordered.

The post-test alerts of all 13 pharmacogenomic CDSS presented a problem definition to the physician, which included the presentation of the raw genotype in the pharmacogenomic CDSS. Furthermore, the medication ordered was part of the problem definition in two pharmacogenomic CDSS [8, 24, 32, 33]. The interpretation of pharmacogenomic test result (phenotype) was included in each problem definition. Beside the phenotype presentation, all pharmacogenomic CDSS also indicated the clinical impact of a potential drug-gene interaction in their interpretation of the results. In the GPS project, a colored traffic signal system informed the physicians of the different pharmacogenomic risk levels when prescribing a drug [22, 23]. Red lights signified drugs with a high risk of severe adverse drug reactions, yellow lights signified drugs with an increased pharmacogenomic risk (caution), and green lights signified drugs with a favorable pharmacogenomic association.

For all 13 included pharmacogenomic CDSS with post-test alerts, a recommendation section is described in the included literature. Within this recommendation, options for a dosage-adjusted medication based on the pharmacogenomic results were offered in seven pharmacogenomic CDSS [19, 20, 23–25, 28, 34–36]. The recommendation further included contraindication and caution factors in one out of the seven CDSS [36]. Four additional pharmacogenomic CDSS advised to select an alternative medication without dosage-adjustments [8, 9, 32, 33] or recommended a drug or dose modification [12, 24, 31]. Drugs which should be avoided were displayed in the recommendation section of three pharmacogenomic CDSS [25, 30, 35]. For the RIGHT No. 1 project, a recommendation section was mentioned but not specified or further described [29].

Override options for the post-test alert were described in the included articles for ten pharmacogenomic CDSS [8, 9, 12, 19, 20, 24, 28–33, 36, 37]. The prescribing physician could choose to continue the order or cancel the prescription in all of them but one (RIGHT (No.3) project). Another override option for prescriptions in the same nine pharmacogenomic CDSS was the modification of the initial drug order. If the physician continued with the order, a second screen prompted the physician to enter a pre-defined reason for overriding the alert. According to Manzi et al., physicians of the GPS project could also enter a free-text reason for overriding the alert [28].

Links to further information were described for almost all (pharmacogenomic CDSS with pre-test alerts except for the CDSS of the GPS project. Each post-test alert of the 12 pharmacogenomic CDSS referred physicians to additional information about related genes or drugs. Six of them also linked the physicians to full guideline texts and original references [11, 19, 20, 24, 25, 32, 33, 35, 37, 38].

Another object of the post-test alerts of five pharmacogenomic CDSS was a note with contact information of a clinical pharmacist [28, 30, 32, 33, 36, 39]. The post-test alert of the GPS project was designed to be read within 30 s or less [28, 34]. In the PMP No. 2 project, the raw genotype results were also presented to the physician when a pharmacogenomic test was ordered redundantly [36]. The information of the post-test alerts of the Warfarin Dosing prototype was arranged in four tabs for structural organization [24].

#### Evaluation

In the last 5 years, the alert design of the UW-PowerChart prototype was evaluated three times within simulated environments. First, Devine et al. evaluated the alert design with seven cardiologists and three oncologists. The participants’ median rating of the alerts usability was two on a Likert scale ranging from one (strongly agree) to seven (strongly disagree). Some physicians suggested minor improvements to the CPOE user interface and the alert design [38]. Second, Overby et al. evaluated the alert design with 15 oncologists and seven cardiologists. Despite a majority (94%) of physicians believing in a relative advantage of pharmacogenomic CDSS, in general only 28% of physicians found the pharmacogenomic CDSS alerts to be useful. Some physicians suggested minor improvements to the CPOE user interface and the alert design [11]. Third, Nishimura et al. evaluated the alert design with 52 physicians. The pharmacogenomic CDSS interface was appropriate for 87% of the participants. Most of the participants reported the alerts to be useful (92%) and at the right time (91%). Furthermore, 80% of the participants preferred an option to prescribe the recommended medication within the alert. Some physicians suggested that supplementary information should be provided as external links [33].

In a study by O’Donnell et al. the use of the Genomic Prescribing System (GPS) alerts was evaluated. During their study period participating patients visited the clinic 268 times. At 86% of the visits, physicians accessed the pharmacogenomic CDSS and received 367 post-test alerts for medications of patients. Physicians clicked on the link within the alert to obtain clinical details when the alert was indicating a drug with a high risk (in all cases) or a drug that should be taken with caution (72% of all cases) [22].

In the RIGHT No.2 project the impact of using alerts to drive providers toward online pharmacogenomics education was limited [35].

The pre-test alerts of the pharmacogenomic CDSS of the PG4KDS project were issued electronically 1106 times and the post-test alerts were issued 1552 times during the study period. Physicians changed their initial prescription for 95% of the patients for whom the alerts provided treatment recommendations [12]. In a study by Gammal et al. six patients with high-risk CYP2D6 genotypes were initially prescribed codeine. Due to a post-test alert during the order process, the prescriber changed the order to a recommended alternative analgesic [31].

Six simulated patient scenarios were designed to evaluate the alert design of the Warfarin Dosing prototype. Twelve participating physicians were required to initiate and adjust warfarin treatments for simulated patients. Overall, participants believed the alerts were useful and well designed. However, they also expressed both some confusion when presented with a warfarin drug-drug interaction alert for medication not included in the pharmacogenomic CDSS and some concerns about ordering an initial dose greater than the standard starting dose. Therefore, some alert design changes were suggested by the participating physicians [24].

### Reports

#### Designs

In 12 pharmacogenomic CDSS pharmacogenomic reports were generated either in the form of summary reports for physicians [10, 18–22, 26–28, 30–32, 34, 39–41] or in the form of patient letters for the respective patients [31, 39, 42]. These reports were either generated as HTML pages [10, 18, 22, 30, 34] or as PDF documents [32, 34]. The reports are presented to the physician either in the local EHR - if the pharmacogenomic CDSS was integrated into the EHR - or in an online portal - if they are not integrated into the EHR. For the pharmacogenomic reports of three pharmacogenomic CDSS a general information section including information such as patient data, sample number, current medication or the ordering physician was described in the articles [21, 28, 41].

Similar to the pre-test and post-test alerts, the reports included a problem description in all 12 pharmacogenomic CDSS, which contained a genotype results section and a result interpretation (phenotype) section. Additionally, in the result interpretation section of six pharmacogenomic CDSS the clinical impact of potential drug-gene interactions was explained [10, 20, 26, 31, 39–42]. The GeneSight system uses a colored traffic signal system, which informs physicians of the different pharmacogenomic risk levels when prescribing a drug. Red lights signify drugs with a high risk of severe adverse drug reactions, yellow lights signify drugs with an increased pharmacogenomic risk (caution), and green lights signify drugs with a favorable pharmacogenomic association [21]. Similarly, in the YouScript system the results were severity-coded (change, consider, monitor, no change) [41].

For seven of the 12 pharmacogenomic CDSS with pharmacogenomic result reports, a recommendation section was mentioned in the articles [10, 18, 26, 28, 31, 39–41] but only for five of them a further description could be found in the included literature [10, 18, 26, 31, 39, 41]. Within these five pharmacogenomic CDSS, dosage-adjusted medication options based on the pharmacogenomic results were recommended. Drugs which should be avoided were displayed in the recommendation section of three pharmacogenomic CDSS.

In the patient letter of the CPMC project, patients were advised to discuss the pharmacogenomic results with their physicians whenever they were prescribed a medication which was affected by these results [42].

Further sources of information were provided by five pharmacogenomic CDSS [10, 28, 31, 39, 40, 42]. Each report of these CDSS referred physicians or patients to additional information about related genes or drugs [28, 31, 39, 40] or to full guideline texts and references [10, 40, 42]. Another object of the pharmacogenomic report in the PG4KDS project was a note with contact information referring to a clinical pharmacist [31, 39].

#### Evaluation

Blagec et al. evaluated the perception and usability of the interactive HTML reports of the MSC pharmacogenomic CDSS amongst 28 physicians and 11 pharmacists [10]. According to Blagec et al. the MSC system was usable (Cronbach’s alpha 0,8), trustworthy (Cronbach’s alpha 0,7) and useful (Cronbach’s alpha 0,7). The results of the workflow integration subscale were heterogeneous. Most of the participants were able to solve the presented patient scenarios with the recommendations displayed on the pharmacogenomic CDSS interface. Participants frequently requested specific listings of alternative drugs and concrete dosage instructions. A common problem among the participants was the negligence of other patient-specific factors such as co-medications or co-morbidities when choosing a drug based on the pharmacogenomic CDSS recommendations [10].

O’Donnell et al. evaluated the pharmacogenomic summary reports of the Genomic Prescribing System (GPS) by surveying 60 physicians. Eighty-six percent of the participants reported that the provided pharmacogenomic summary reports were clinically relevant (“strongly agree”: 10%, “somewhat agree”: 76%) [22].

Laerum et al. evaluated the pharmacogenomic CDSS reports of the genAP project in a simulated environment with nine participating physicians. All physicians rated the contents of the pharmacogenomics CDSS as useful, trustworthy, clearly presented and worthwhile logging into the system. Nevertheless, the explanation of the report algorithm led to considerable confusion amongst the participants. On average, physicians needed more than 40 s to study and understand the report structure. According to Laerum et al., background information and references should be included in the reports, but less prominently displayed than the results and recommendations [40].

Brixner et al. reported that the physicians’ satisfaction with the pharmacogenomic CDSS reports of the YouScript system was high. Ninety-five percent of all physicians considered the pharmacogenomic CDSS to be helpful for clinical decision-making. However, it might be important to mention that the software company Genelex (vendor of YouScript) supported this study by providing buccal swab collection materials, shipping, genotyping and support for the pharmacogenomic CDSS (YouScript) report [41].

### EHR data

#### Designs

Another feature of ten pharmacogenomic CDSS was the ability to store pharmacogenomic results in the EHR. In four pharmacogenomic CDSS the generated pharmacogenomic reports were stored in the EHR and made available to physicians during clinical routine [19, 20, 30, 34, 39] (four of them stored summary reports in the lab section [19, 20, 30, 34] and one did so with patient letters [39]). Another CDSS provided a hyperlink in the lab section to display the content of the pharmacogenomic report in PDF format [42].

Raw pharmacogenomic results in form of genotype results were stored in the lab section of the EHR in eight pharmacogenomic CDSS [9, 19, 28, 30, 32–35, 39, 43]. One of them stored not only genotype results relevant to the patients’ current medication, but all available pharmacogenomic test results, to get an overview of all pharmacogenomic tests ordered for this patient so far [20]. Three out of eight pharmacogenomic CDSS entered the results in a special problem list within the lab section for a better overview [28–30, 34].

Four out of eight pharmacogenomic CDSS also provided a phenotype interpretation in the EHR [19, 20, 30, 32, 39], three of them in the lab section [19, 20, 32, 39]. Beside phenotype interpretation, two pharmacogenomic CDSS further explained the clinical significance of the pharmacogenomic test results in the EHR [30, 32].

In one pharmacogenomic CDSS dosage-adjusted medication options which were based on pharmacogenomic test results were also stored in the EHR [43]. Goldspiel et al. mentioned in their article that override reasons would be passed on to the medication order tracking field, to communicate this information to other care providers when reordering, verifying, dispensing or administering this medication [9].

#### Evaluation

Overby et al. evaluated the pharmacogenomic information stored in the EHR in a simulated environment with 15 oncologists and seven cardiologists. Fifty percent of participating physicians believed, that the genetic test results within the EHR laboratory section were useful. Some physicians suggested minor improvements of the user interface of the UW-PowerChart prototype [44].

According to Peterson et al. the pharmacogenomic CDSS designs of the PREDICT project were accepted by the participating clinicians and the Pharmacy and Therapeutics committee. However, subsequent changes were required. For the laboratory section of the EHR, participants preferred to display any pharmacogenomic test result, whether indicating a relevant gene variant or not. This might enable physicians to determine, if a patient has already been tested or not [20].

### Inbox messages

When a new entry was added to the problem list in the EHR [39] or a new pharmacogenomic test result was either available in the EHR [9, 29, 35, 36, 43] or in the web portal [42], an automated inbox message was sent to a physician or nurse practitioner in nine pharmacogenomic CDSS [9, 29–31, 35, 36, 39, 42, 43]. The inbox messages contained patient data such as the patient’s name, medical record number and pharmacogenomic data, such as gene name and phenotype interpretation. In one pharmacogenomic CDSS, links to additional information about related genes, full guideline texts and original references were provided [35]. Another feature of two pharmacogenomic CDSS was a pharmacogenomic consultation for physicians. Physicians could request a consultation through an EHR message to a pharmacist. The following consultation took place either virtual in form of a text message response or in person [22, 30].

### Search engines

Search engines were part of two pharmacogenomic CDSS [18, 22, 23]. They enabled prescribers to consider pharmacogenomic information when comparing drug treatments for given clinical indications. In the GPS project the search results were tabulated. Furthermore they were displaying the chosen drug, the related pharmacogenomic risk level in form of potential gene-drug interactions and the level of evidence for this drug as an appropriate treatment for the disease [22, 23]. Additionally, links to additional information were provided. In the MSC system a physician could use the search engine in two different ways [18]. First, he or she selected a medication and a known genetic variant of the patient to receive recommendations on drug dosing or alternative treatment. Second, he or she could select a medication and see all possible recommendations, which were programmed into the MSC system and which could potentially be displayed to a physician within a report.

### Others

#### Designs

The MSC system provided an interface to upload a patient’s genetic profile from 23andMe or VCF (Variant Call Format) files to generate a MSC QR (Quick Response) code. This interface comprised both an instruction to guide the upload process and to select a file format of strand orientation, and a file uploader tool. The generated QR codes were printed on both paper-based lab reports for physicians and personalized cards for patients to transport and use the genetic information for clinical decision support [18].

If a drug was ordered in the PGTIC project, the status of the drug-related pharmacogenomic test was displayed on the order form (whether the result was present, absent, pending, or whether the test has not yet been ordered). Furthermore, order options were provided in the form of predefined override options or in the form of a notice that the prescription ordering has been blocked [9].

Furthermore, in the pharmacogenomic CDSS of the RIGHT (No. 3) and the PREDICT project, an online portal for patients was included. Patients could access their pharmacogenomic test results via this online portal [19, 20, 25].

#### Evaluation

According to Peterson et al. 2013, the online patient portal of the PREDICT project was deemed to be useful. Patients stated that they preferred detailed and descriptive background information about their genotype-related risk of side effects [20].

## Discussion

Within the last 5 years, several pharmacogenomic CDSS have been developed which have the potential to support the incorporation of pharmacogenomic testing into clinical routine. They comprise different forms of active and passive CDS targeting both physicians and pharmacists. However, most of the included articles are solely describing prototypes of pharmacogenomic CDSS rather than evaluating them.

We performed a literature research to conduct a scoping review of the designs of user-system interactions of pharmacogenomic CDSS. We limited our PubMed search to the articles published between January 1, 2012 and November 30, 2016 to focus on pharmacogenomic CDSS which were recently developed and published. Nevertheless, we might have neglected relevant designs of user-system interactions published before 2012.

We found pre-test and post-test alerts to be amongst the most cited user-system interactions within recently published pharmacogenomic CDSS. As mentioned by Bell et al. pharmacogenomic test results remain relevant to the medical treatment of a patient over his/her lifetime, and need to be stored in a way that they will not be forgotten or lost [12].

Recommendations, which are presented in a pharmacogenomic alert, need to be formulated carefully for several reasons. First, other variables besides genetics such as “comorbidities” or “co-medications” should be considered when prescribing drugs [10]. Therefore Manzi et al. refrained from using words such as “should” or “must” and from dictating exact dosing adjustment recommendations [28]. Second, presenting specific drug alternatives or dose adjustment recommendations in an alert might also raise concerns about the liability in case of an adverse drug event, especially when different guidelines are displayed for the same drug [10, 33].

In this context, alert fatigue has been mentioned as another main challenge for using alerts within pharmacogenomic CDSS [25, 28, 38]. To avoid over-alerting caused by repetitive alerts Ji et al. [25] included exclusion criteria in the rules and Manzi et al. [28] designed their alerts to only notify if an action is recommended by the physician. Both strategies should be considered and combined when implementing alerts into pharmacogenomic CDSS.

Overcoming technical barriers seems to be another main challenge in designing alerts for a pharmacogenomic CDSS. For instance, Nishimura et al. intended to link physicians directly from the alert to the patient’s pharmacogenomic lab results in the local EHR. In their study, such a link was considered to be useful for user-system interactions. However, due to restrictions of the vendor-based CDSS it was impossible for Nishimura et al. to integrate such a link into the post-test alerts [33].

Only four alert designs of pharmacogenomic CDSS have been evaluated since 2012 and were described in seven articles. Three studies relating to two pharmacogenomic CDSS demonstrated that alerts can lead to a change of the initial prescription and therefore prevent severe adverse drug events effectively [12, 22, 31]. These order changes can be seen as the physician’s acceptance of the pharmacogenomic CDSS alert. Furthermore, the physicians’ acceptance of the UW-PowerChart prototype alerts has been high [11, 33, 38]. In contrast, using alerts to drive providers towards online pharmacogenomics education might be ineffective [35].

The acceptance of pharmacogenomic CDSS reports was high amongst those physicians and pharmacists who participated in the four studies that evaluated four different pharmacogenomic CDSS [10, 22, 40, 41].

Delivering pharmacogenomic information not only to physicians but also to patients might be a crucial feature of pharmacogenomic CDSS. Pharmacogenomic information is usually gathered within the environment of a particular clinic or health care provider. Via patient letters [18, 31, 39, 42] and online portals [25], which contain the pharmacogenomic test results, this information can be transferred to other health care providers. For instance, patients can be advised to discuss their pharmacogenomic test results with physicians whenever a medication is prescribed which is affected by these pharmacogenomic results [42]. In the evaluation study by Peterson et al. the online patient portal of the PREDICT project was deemed to be useful and was accepted by participating patients [20].

Another essential function of many pharmacogenomic CDSS was storing pharmacogenomic information in the EHR. For instance, if override reasons for alerts are passed on to the order tracking field of a medication, they can be communicated to other care providers when they reorder, verify, dispense or administer this medication [9]. Furthermore, the storage of all pharmacogenomic test results (not only of the relevant ones) in a patient’s medical record offers physicians an overview of all pharmacogenomic tests which have already been carried out. As a result, reordering the same pharmacogenomic test might be avoided [20]. Collecting all pharmacogenomic information in a separate problem list within the laboratory section might also serve as an option for a quick overview of all potential gene-drug interactions known for a particular patient [28–30, 34]. 11 out of 22 physicians who participated in the evaluation study by Overby et al. believed that the genetic test results within the EHR laboratory section were useful [44].

Via inbox messages physicians can be informed about new pharmacogenomic test results, which are available within the laboratory section of a patients’ medical record [9, 29, 31, 35, 36, 39, 42, 43]. If the physician needs any kind of advice regarding the pharmacogenomic test results, he/she might request a consultation by sending a message to a clinical pharmacist [22, 30]. This provides the physician with an active decision support before he/she orders a particular medication at the point-of-care.

With a search engine physicians are enabled to search for a disease indication and compare various medical treatment options based on a patient’s pharmacogenomic information. The benefit of such a user-system interaction is that physicians can get the decision support before they decide which medication they want to prescribe. In contrast, the use of alerts requires the physician to first select a medication before getting the pharmacogenomic information in response [18, 22, 23].

Recommendations for the implementation of a particular design of user-system interaction can only be made on a very high level since the recently developed pharmacogenomic CDSS have not been sufficiently evaluated yet within a clinical setting. Implementing pre-test or post-test alerts seemed to be the most popular approaches. However, such active CDS tools require a comprehensive and well-curated pharmacogenomic knowledge base. Such a knowledge base has to be both developed and maintained by physicians with sufficient knowledge of pharmacogenomic CDSS prior to the implementation of pharmacogenomic alerts. We recommend the establishment of both the necessary group of medical experts and the corresponding knowledge base in order to further evaluate pharmacogenomic alerts.

However, we recommend the implementation of a passive CDS in the form of structured pharmacogenomic reports in the first instance. Clinical reports for clinicians have been well-established in clinical environments over many years [45–47] even though such reports might be unstructured or incomplete in some cases [48, 49]. Therefore, we believe that the implementation of a structured pharmacogenomic report would most likely fit into the working habit of a clinician.

Future pharmacogenomic CDSS will likely include prediction models to recommend pre-emptive genotyping for patients exceeding particular risk thresholds. A patient’s diagnosis might be, contain or induce a risk factor, which will likely require a medication with known gene-drug interactions within the next few years. Whenever such a diagnosis is entered into a patient’s medical record, an alert might be set off, which recommends pre-emptive genotyping [50, 51]. This will further enhance the usefulness of integrating alert and inbox message options into a pharmacogenomic CDSS. Prediction models have previously been used, but only to identify potential patients for a pharmacogenomic CDSS study [20, 25]. Nevertheless, risk thresholds or risk scores should be defined carefully and in consensus with the medical staff in charge. Otherwise, inadequate risk thresholds might lead to over-alerting and alert fatigue.

A limitation of this study is its methodological rigor as compared to a full systematic review. This is especially relevant to the selection of the source databases. We only used the MEDLINE Database, which might limit the completeness of our search. However, we believe that our adherence to high methodological standards throughout this review as defined in the PRISMA statement helps to minimize bias on study selection and reporting of evidence.

We used the keywords “pharmacogenetic*”, “pharmacogenomic*”, “decision support” and “medical decision making” for the search query. These are the most common terms in literature to describe a pharmacogenomic CDSS. It is possible that these keywords did not cover all pharmacogenomic CDSS published since 2012. In our opinion, using the most common terms was sufficient to provide a broad range of the designs of user-system interactions which were used in recently published pharmacogenomic CDSS. To conduct a systematic review of this topic, an advanced search query with further relevant terms might be preferable

We did not assess the risk of bias. The reader therefore cannot assess the validity of the individual studies included in this scoping review.

As this review is intended to describe the functionality and designs of user-system interactions of pharmacogenomic CDSS, it is also limited in collecting evidence for the effectivity and the overall medical usability of CDSS for pharmacogenomics in the clinical setting.

Evaluating the users’ acceptance of designs of user-system interactions was part of only a few articles in our scoping review. Further evaluation efforts addressing this topic will be necessary will be necessary to support the development of prototypes.

## Conclusion

Pre-testing and post-testing alerts seem to be the most relevant designs to physician-system interactions in the 20 pharmacogenomic CDSS. Such alerts need to be implemented carefully to prevent alert fatigue and to avoid liabilities. In addition, pharmacogenomic test results and override reasons which are stored in the local EHR can help to communicate pharmacogenomic information to other internal care providers. Integrating patients into user-system interactions via patient letters and online portals are crucial for transferring pharmacogenomic data to external health care providers. Furthermore, inbox messages can inform physicians about new pharmacogenomic test results and enable them to request pharmacogenomic consultations. Search engines enable physicians to compare medical treatment options based on a patient’s genotype. To support the development of prototypes further evaluation efforts regarding the designs of user-system interactions will be necessary. In the future, pharmacogenomic CDSS will likely include prediction models to identify patients who are suitable for preemptive genotyping.

## Additional files

Additional file 1: PRISMA checklist for the process of searching and selecting articles. Information is provided for 22 out of 27 points recommended. (DOC 63 kb)

Additional file 2: In-depth overview of the features of the selected pharmacogenomic CDSS. Microsoft Excel spreadsheet format (.xls); providing an overview of the features of the 20 selected pharmacogenomic CDSS. (XLS 60 kb)

## Abbreviations

CDS: Clinical decision support
CDSS: Clinical decision support systems
CPMC: Coriell personalized medicine collaborative
CPOE: Computerized physician order entry system
CPS: Clinical pharmacogenomic service
EHR: Electronic health record
GPS: Genomic prescribing system
HIS: Hospital information system
MSC: Medicine safe code
PGTIC: Pharmacogenetics testing implementation committee
PMP: Personalized medication program
QR: Quick response
s/n: surrogate name
VCF: Variant call format
